# Draughts and Deaths

**Published:** 1893-06

**Authors:** William Chambers


### DRAUGHTS AND DEATHS.

Many old people, as well as persons in middle life, are subject to rheumatism, a species of pain or disease, which, like the toothache, meets with little general sympathy, because it is not frequently immediately fatal in its attacks. In the case of many who belong to professions where exposure to atmospheric changes from heat to cold, and dry to wet, necessarily takes place, it is almost impossible to prevent rheumatism occasionally taking effect ; but in not a few instances this painful malady might be avoided, simply by being a little more careful of our persons.

There are some people, who, because they are stout and healthy, and have good appetites, and have hardly ever been ill all their days, think that they may do anything with themselves, and therefore cherish the dangerous idea that "they will not kill." Whenever we see people of this description, we are afraid of them. We know, from experience, that it is they who have the chance of being cut off first among our acquaintances, and so look upon them as persons, who, braving death at every corner, will some day soon be numbered with the dead.

On the other hand, we have never any fears for the man who is always complaining of something trifling being the matter with him; for we know that he takes good care of himself, and, like a creaking hinge, he will endure a great deal before he parts with existence. People of this sort are dreadfully jealous of an open window, or a broken pane of glass, or a door standing ajar, and well they may, for it is at these holes that rheumatisms, colds, coughs, consumptions, and deaths, get admittance and surprise the inmates. There may be often something ludicrous in the fears excited by seeing the openings in windows and doors which we mention; but we would advise all who prefer good to bad health, and a warm bed to a place in the churchyard, to submit to any kind of ridicule, rather than sit down in a room, a church, a coach, or any other place in which there is a draught of air playing about, and seeking whom it may devour. If they be wise, they will either see the opening which causes the said draught, closed, or at once make good their retreat. Better to leave the company, and all its fascinations, sound in lith and limb, than have the chance of retiring with at least a rheumatic pain in the shoulder, which sticks to you for years, and seems as if you were perpetually enduring the cut of an axe or the boring of an awl in your flesh and bones.

We are convinced that many young persons literally kill themselves out of mere carelessness and bravado. We have a distinct remembrance of a fine, tall, stout, gentlemanly man of our acquaintance, thus committing a suicide, He measured six feet, three inches in height, was well built in body; and when he shook any one by the hand, it was like the grip of a vice. He was a true Hercules in frame; and on looking at him as he paced along the pavement with graceful ease and stateliness, you would have been inclined to say, there goes a man who will live many years: death will find it no easy matter to bring him down. Such a fallacy! We saw him one fine sunshiny day walking on Prince's street, and none could be compared with him in point of appearance; people turned about and looked at him as he passed. Six days elapsed, and he was lying in his grave. Some business or pleasure had called him a short distance into the country. In coming back, he had missed the stage which he expected would convey him back to town. But this was no disappointment; he was fond of a journey on foot; what was a few miles to him? So he walked home, and overheated himself; took off his shoes and sat for a few minutes in a draught before an open window. In an instant of time he caught his death. A short cough: a creeping cold all over the body: inflammation in the breast, or lungs—it is all one: the doctor: bleeding: high fever: death: the undertaker: funeral letters: and the churchyard. Such was the routine of destruction in the case of perhaps the handsomest man that ever walked on the streets of Edinburgh. Will his example serve as a warning?

We are ever complaining of being affected with colds, and coughs, and rheumatisms, and other diseases, yet we seem to take little care in preventing their intrusion. One-half of the deaths which occur are brought about by our own follies, or our own carelessness. Because we are well, we think we shall never be ill. We go out to evening parties without great coats, or cloaks, or something warm to wrap around our mouths and necks in coming home. We come out of theatres heated to the suffocating height of eighty and ninety degrees, and plunge into an atmosphere almost at the freezing point, and that without a fear of the consequences. We are also criminally careless about the state of our feet. We walk about in wet weather, and come home with damp shoes or boots—will not be at the least pains to change them for others which are dry and comfortable. Of course, colds and coughs ensue; perhaps, also, we procure ourselves some smart twinges in the stomach, and administering a dram by way of antidote, probably hasten an incipient inflammation to its crisis. There is not one of our readers who cannot recall instances of deaths among his acquaintances, caused in this or a similar manner.—*William Chambers.*

